# Levels of soluble fms-like tyrosine kinase one in first trimester and outcomes of pregnancy: a systematic review

**DOI:** 10.1186/1477-7827-9-77

**Published:** 2011-06-08

**Authors:** Marni Jacobs, Natasha Nassar, Christine L Roberts, Ruth Hadfield, Jonathan M Morris, Anthony W Ashton

**Affiliations:** 1 Perinatal Research, Kolling Institute of Medical Research, University of Sydney, Royal North Shore Hospital, St Leonards, 2065, NSW, Australia; 2 Department of Obstetrics and Gynecology, Royal North Shore Hospital, St Leonards, 2065, NSW, Australia

## Abstract

Angiogenic factors are involved in formation of new blood vessels required for placental development and function; and critical for fetal growth and development. Soluble fms-like tyrosine kinase 1(sFlt-1) is an anti-angiogenic protein that inhibits formation of new blood vessels resulting in potential pregnancy complications. The objective of this study was to undertake a systematic review to assess levels of sFlt-1 in early pregnancy and association with adverse pregnancy outcomes. PubMed and Medline databases and reference lists were searched up to July 2010. Inclusion criteria were pregnant women, blood sample taken during first trimester and assessment/reporting of sFlt-1 concentrations and subsequent pregnancy complications. Twelve relevant studies were identified of 71 to 668 women. No pooling of results was undertaken due to variation in sFlt-1 concentrations (range, 166-6,349 pg/ml amongst controls), samples used (serum, plasma), different summary statistics (mean, median, odds ratio) and outcome definitions applied. Levels of sFlt-1 were generally higher among women who developed preeclampsia (11 studies) or gestational hypertension (two studies), but not significantly different to normotensive women in most studies. There was no consistent pattern in association between sFlt-1 concentrations and fetal growth restriction (4 studies); and levels were non-significantly higher for women with postpartum bleeding (1 study) and significantly lower for stillbirths (1 study).This review found no clear evidence of an association between sFlt-1 levels in first trimester and adverse pregnancy outcomes. However, findings were affected by methodological, biological and testing variations between studies; highlighting the need for consistent testing of new biomarkers and reporting of outcome measures.

## Background

Despite appropriate antenatal care, unforeseen pregnancy complications still arise. Adverse pregnancy outcomes such as preeclampsia, fetal growth restriction and stillbirth may share the common basis of abnormal placentation [1,2]. Placental development is facilitated by the coordinated and complex processes of vasculogenesis (*de novo *formation of blood vessels) and angiogenesis (formation of new blood vessels from pre-existing vessels), both prior to implantation and throughout gestation. Impairment of these processes may result in restricted blood flow to the fetus, increased maternal blood pressure and premature delivery [1-3].

Biomarkers of angiogenesis are proposed as potential predictors of adverse pregnancy outcomes [1-3]. Soluble fms-like tyrosine kinase (sFlt-1) is an anti-angiogenic protein and considered to be one of the most promising serum biomarkers identified to date [4,5]. sFlt-1 inhibits vascular endothelial growth factor and placental growth factor signaling, attenuating the formation of new blood vessels and promoting maturation of those vessels that have already been created. sFlt-1 has been implicated in the pathogenesis of preeclampsia when infusion of recombinant protein into rodents produced many of the symptoms of this enigmatic disease [6].

Although increases in sFlt-1 concentrations are expected during the second and third trimester of pregnancy, some studies have found rapidly increasing levels of this protein to arrest placental development, resulting in pregnancy complications [4,5]. In particular, elevated levels of sFlt-1 have been reported in the second and third trimesters among women with pregnancies complicated by preeclampsia compared to women with normal pregnancies [5,7,8]. Increased sFlt-1 expression in the maternal circulation during late pregnancy has also been found to occur among women with growth restricted fetuses compared to those with normally grown infants [9,10], and in women with placental abruption; although results are inconsistent [11,12].

Levels of anti-angiogenic proteins can be detected soon after conception, and may provide a means of screening for women at risk for developing these complications [13,14]. Most studies of sFlt-1 concentrations and pregnancy outcomes have been conducted late in pregnancy or at birth with timing of the results inadequate for use as a predictive screening tool. The potential advantages of first trimester screening include the opportunity to incorporate an additional test into existing, routine antenatal testing and identification of at-risk pregnancies at gestations when preventive interventions may be a realistic option. The aim of this study was to undertake a systematic review of published studies to determine whether 1^st ^trimester sFlt-1 concentrations are associated with adverse pregnancy outcomes.

## Methods

### Data Sources

We identified relevant studies by searching PubMed and Medline databases to identify publications up to May 2011, using the terms: 'Flt-1' or 'VEGFR-1' in conjunction with one of the following: 'pregnancy', 'adverse pregnancy outcomes', 'pregnancy complications', 'stillbirth', 'preterm birth', 'prematurity', 'small for gestational age', 'SGA', 'IUGR', or 'preeclampsia'. Search limits were set to exclude non-human studies, research among men, or articles published in a language other than English. We also reviewed the reference lists of identified articles and relevant review articles on the subject to identify studies that may have been missed in the initial database search.

### Study Selection

The criteria for inclusion in the present systematic review were any published studies which: i) investigated the relationship between sFlt-1 concentration in the blood (serum or plasma) of pregnant women during the first trimester (mean specimen collection prior to 14 weeks gestation) and subsequent pregnancy complications, and ii) provided mean or median measurements of sFlt-1 concentrations with standard deviations, risk estimates with 95% confidence intervals, or data permitting to the calculation of these. Case-reports, letters, and review articles were excluded from the current investigation. Pregnancy outcomes were pre-specified, and included: preeclampsia, stillbirth, preterm birth, small for gestational age (SGA), and intrauterine growth restriction (IUGR).

Two reviewers independently assessed each study for inclusion in the review. Data were independently extracted from each paper by two reviewers onto a standard data extraction form. Discrepancies were resolved by consensus. Studies that met the inclusion criteria were assessed for: location of study, study design, study population, gestational age at screening, exposure assessment, sample size, and reported effect measure.

### Statistical Analysis

We planned to undertake a meta-analysis of continuous data and apply the mean difference approach for each outcome where threshold levels of sFLT-1 were reported [15]. However, we were unable to pool results because there was significant variation in the selection of study populations, the quantification of sFlt-1 (evidenced by large variations in the reported levels), the samples used (serum or plasma), the choice of summary statistic (mean, median and OR) and outcome definitions. Thus, the general findings from the studies are reported and the percentage difference (positive or negative) in sFLT-1 levels among women with or without adverse outcomes was calculated.

### Ethics

No ethics approval was required.

## Results

**Figure 1:**
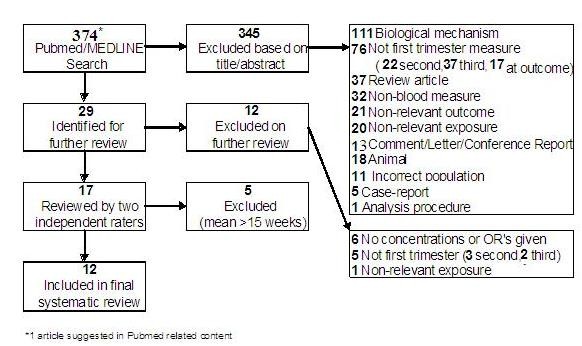
**Identification of studies for inclusion in the systematic review**.

The primary search yielded 373 articles; and one additional paper was identified through PubMed-suggested related content. After screening the titles and abstracts of these studies, 29 publications were identified for further review based on stated inclusion criteria. Ultimately, twelve studies were included in the present analysis [16-27]. Figure 1 depicts the selection process, including reasons for the exclusion of identified publications. Examples of non-relevant outcomes identified were cancer, chronic lung disease, arthritis, and peripheral artery disease. Non-relevant exposures that were assessed included angiogenic proteins other than sFlt-1 (such as sEng and VEGF), non-relevant markers (such as IL-8 and IL-6), and lifestyle factors (such as smoking).

The characteristics of the twelve studies included in the final review are presented in Table 1. Screening was performed between 4 and 17 weeks gestation with mean age of gestation at screening ranging between 9 and 13.7 weeks. The majority of studies identified utilized a case-control study design with some studies nested within and based on serum samples and data collected as a part of larger randomized trials or cohort studies. There were two prospective cohort studies that included women attending for prenatal care [25,26]. One of these studies also included women from an obstetric medicine clinic [26].

**Table 1:** Characteristics of studies assessing sFlt-1 in first trimester of pregnancy

Reference	Location	Study Design	Type of sample and gestational age at screening	Participants	Outcome(s)	s-FLT1 Summary measure
Akolekar, 2010	United Kingdom	nested case-control	Plasma 11-13 weeks	Singleton pregnancies Exclusions: hypertension but uncertainty about a diagnosis of preeclampsia (n = 30), preeclampsia but stored blood not available (n = 57)	Preeclampsia < 34 weeks ≥ 34 weeks	median
Baumann, 2008	Switzerland	case-control	Serum 11-14 weeks	Singleton pregnancies Exclusions: women with history of hypertensive disorders, immunological diseases, HELLP, or PE < 34 weeks	Preeclampsia	mean, median
Chaiworapongsa, 2005	Chile	case-control	Plasma 7-16 weeks	Singleton pregnancies. Exclusions: pregnancies with fetal anomaly or demise, vaginal bleeding, women with serious medical conditions, chronic hypertension, asthma requiring medication, or requiring the use of anti-platelet or non-steroidal anti-inflammatory drug.	Preeclampsia	mean
Erez, 2008	Chile	nested case-control	Plasma 6-15 weeks	Singleton pregnancies.	SGA, Preeclampsia (preterm and term)	median, OR
Eskild, 2008	Norway	nested case-control	Serum 4-12 weeks	Normotensive pregnant women only.	Excessive postpartum bleeding	mean
Lynch 2010	USA 2005-2008	prospective cohort	Serum 10-15 weeks	Singleton pregnancies, women presenting for prenatal care Exclusions: intrauterine fetal loss, gestational hypertension	Preeclampsia	mean
Noori 2010	United Kingdom	prospective cohort	Serum 10-17 weeks	Singleton pregnancies from routine screening (n = 114) and an obstetric medicine clinic (n = 45)	Preeclampsia, (preterm and term) Gestational hypertension	geometric mean
Rana S, 2007	USA, 2001-2003	nested case-control	Serum 11-13 weeks	Singleton pregnancies delivered > 20 weeks (livebirths) gestation. Exclusions: women with history of hypertension, hypertension 6 weeks postpartum, renal disease, or diabetes.	Preeclampsia,	mean
Smith, 2007	Scotland, 1997-99	nested case-control	Serum 10-14 weeks	Singleton pregnancies. Exclusions: stillbirths due to congenital anomalies or rhesus disease.	Preeclampsia SGA Preterm (extreme and moderate) Stillbirth	OR
Thadhani R, 2004	USA, 2001-2003	nested case-control	Serum 8-14 weeks	Singleton pregnancies > 20 weeks gestation. Exclusions: women with hypertension > 6 weeks postpartum, history of thyroid, liver, or chronic renal disease, or chronic hypertension.	Preeclampsia, Gestational hypertension SGA	mean
Vatten LJ, 2007	Norway, 1992-1994	nested case-control	Serum 4-12 weeks	Singleton pregnancies	Preeclampsia (preterm and term)	mean, OR
Wathen KA, 2006	Finland, 2001-2002	longitudinal case-control	Serum 2-15 weeks	Singleton pregnancies among Caucasian women Exclusions: women with history of hypertension, diabetes, chronic disease, or smoke > 5 cigarettes/day.	Preeclampsia IUGR	median, OR

All blood testing done using an ELISA testing kit developed by R&D Systems

SGA = small for gestational age; IUGR = intrauterine growth restriction

The outcomes reported included preeclampsia (11 studies) [16-18,20-27], SGA or IUGR (four studies) [18,21,22,24], gestational hypertension (two studies) [22,26], and one study each evaluated excessive postpartum bleeding [19] and stillbirth [21]. Concentrations of sFlt-1 were presented either as mean or median concentrations, or as odds ratios (OR) for various levels of sFlt-1 (continuous, quartiles, or deciles). While assessment of circulating sFlt-1 was performed using an ELISA testing kit (R&D Systems Pty Ltd) according to manufacturers' guidelines, other aspects of testing and specimen storage varied between studies (Table 2). Analysis was performed on either serum or EDTA anti-coagulated plasma. Sample storage temperatures ranged from -20°C to -80°C, and the intra- and inter-assay coefficients varied between laboratories along with sensitivity levels. Only three studies reported the dilution used [16,19,23] and six laboratories conducted duplicate testing [20,22,24-27]. In addition, only one study explicitly reported how long the samples were stored for prior to testing [22], although two others referenced duration of sample storage in their discussion sections [16,23] and another specified that none of the samples had previously been thawed and refrozen [27].

**Table 2:** sFlt-1 testing characteristics

Reference	Storage Temperature	Dilution	Duplicate Testing	Intraassay Coefficient	Interassay Coefficient	Sensitivity
Akolekar, 2010	-80°C		yes			15 pg/mL
Baumann, 2008	-30°C	1:3		3.8%	7.0%	
Chaiworapongsa, 2005	-70°C			6.9%	4.8%	17.8 pg/mL
Erez, 2008	-70°C			3.9%	1.4%	17.0 pg/mL
Eskild, 2008	-20°C	1:5				17 pg/mL
Lynch, 2010	-80°C		yes		9.9%	
Noori, 2010	-70°C		yes			3.5 pg/mL
Rana S 2007	-80°C		yes	3.5%	8.1%	
Smith, 2007				10.4%	14.9%	5 pg/mL
Thadhani, 2004	-80°C		yes	3.5%	8.1%	
Vatten, 2007	-20°C	1:5				17 pg/mL
Wathen, 2006	-80°C		yes	5.0%	8.2%	5 ng/L

All blood testing done using ELISA testing kits developed by R&D Systems.

The outcome most often studied in these investigations was preeclampsia, with standard and consistent definitions used to define preeclampsia in all studies [16-18,20-27]. Investigators typically defined preeclampsia as new onset hypertension (systolic blood pressure ≥ 140 mm Hg or diastolic blood pressure ≥ 90 mm Hg) occurring after 20 weeks gestation, with concurrent proteinuria (≥ 2+ by dipstick or ≥ 300 mg in 24 hours). In some cases, preeclampsia was examined in subgroups based on timing of diagnosis, either preterm (< 34 or < 37 weeks gestation) [18,23,26,27] or term (≥ 37 weeks gestation) [18,26].

The results of the ten preeclampsia studies that reported either mean or median sFlt-1 concentrations are presented in Table 3[16-18,20,22-27]. The concentrations of sFlt-1 varied greatly between the studies with levels in controls (normotensive women) ranging from 166 to 6,349 pg/ml and this did not appear to be related to the gestational age at testing. Eight studies assessed any preeclampsia or preeclampsia at term and reported the mean or median sFlt-1 concentration during the first trimester. Of these, six studies found sFlt-1 levels among women with preeclampsia were 5% to 15% higher compared to women with normal pregnancies [16,17,20,22,24,25]; however, this elevation was only statistically significant in one study [16]. One study of term preeclampsia reported a statistically significant lower concentration (by 19%) among term preeclamptic women [18], while another found non-significant lower concentrations [26]. The findings were similarly variable in the three studies that analyzed preterm preeclampsia (< 37 weeks gestation) separately [18,23,26]. Two studies [18,23], found that concentrations of sFlt-1 were significantly lower (by 19% to 27%) during the first trimester among women diagnosed with preterm preeclampsia [18,23]. However a third study found significant increases in maternal sFlt-1 (by up to 40%) among women with preterm preeclampsia [26] (Table 3). Conversely, no statistically significant increase in the median sFlt-1 concentration among women with preeclampsia at < 34 weeks or ≥ 34 weeks gestation was reported in the final study [27].

**Table 3:** Results from studies assessing the association between 1^st ^trimester sFlt-1 concentrations (pg/mL) and preeclampsia (PE)

Reference	Mean GA	Outcome	Cases (Preeclampsia)		Controls (normotensive)		P-value	% difference†
						
			n	Flt-1 concentration	n	sFlt-1 concentration		
**Studies reporting mean concentrations**				**mean, SD/SE**		**mean, SD/SE**		
Baumann, 2008	12.3	PE (> 34 weeks)	46	1764 (757)	92	1537 (812)	0.04	+13
Chaiworapongsa, 2005	12.3	PE	34	546 (271)	37	464 (260)	0.10	+15
Lynch, 2010	12.3	PE	31	1374 (639)	637	1313 (521)	0.5	+5
Noori, 2010	13.7	PE (< 37 weeks)	10	2414 (622)	128	1729 (85)	< 0.01	+40
		PE (≥ 37 weeks)	10	1688 (210)	128	1729 (85)	0.19	- 2
Rana S, 2007	11-13*	PE	39	3500 (300)	147	3000 (100)	0.14	+14
Thadhani, 2004	10.7	PE	40	1048 (657)	80	973 (490)	> 0.05	+ 7
Vatten, 2007	9.0	Preterm PE	110	135	276	166	0.01	-19
**Studies reporting median concentrations **				**median**		**median**		
Akolekar 2010	11-13*	PE (< 34 weeks)	30	7099	180	6349	NS	+12
		PE (≥ 34 weeks)	60	6840	180	6349	NS	+ 8
Erez, 2008	12.2	PE (< 37 weeks)	17	1308	201	1788	0.03	-27
	12.2	PE (≥ 37 weeks)	39	1448	201	1788	0.02	-19
Wathen, 2006	13.7	PE	44	481	51	432	> 0.05	+10

*No mean gestational age (GA) provided

† % difference in case mean or median 1^st ^trimester sFLT-1 compared with the control level

Two studies also considered gestational hypertension (i.e. hypertension during pregnancy without proteinuria). Thadhani et al found similar first trimester sFlt-1 concentrations in women with gestational hypertension compared to normal controls (942 pg/mL vs. 973 pg/mL respectively, P = 0.52) [22], while Noori et al found significantly higher mean concentrations among those with gestational hypertension (2219 pg/mL vs 1729 pg/mL, P < 0.01) [26].

Four studies investigated maternal sFlt-1 concentrations and IUGR or SGA [18,21,22,24], however, the definition of the outcome varied between studies. Two studies defined SGA as a birth weight below the 10^th ^percentile for gestational age [18,22], while a third defined SGA as a birth weight below the 3^rd ^percentile for gestational age [21]. Wathen *et al*. defined IUGR as birth weight at least 2 standard deviations below the national average for gestational age [24]. There was no consistent pattern in the association between sFlt-1 concentrations and IUGR in the three studies that examined this outcome. In two of the studies [22,24], levels of sFlt-1 among women with growth restricted infants were slightly higher compared with control women (1011 pg/mL vs. 973 pg/mL and 486 pg/mL vs. 432 pg/mL). However, the other study reported lower sFlt-1 concentrations among the SGA group (1616 pg/mL vs 1788 pg/mL) [18].

Two studies examined the dose-response between sFlt-1 levels and size at birth with conflicting results [18,21]. Erez *et al*. treated sFlt-1 as a continuous variable in a polytomous logistic model, adjusted for maternal age, BMI, and nulliparity; and found that compared to normal pregnancies, there was no association between increasing levels of sFlt-1 and delivery of an SGA neonate (OR 1.00; 95%CI 0.99-1.00) [18]. In contrast, Smith *et al*. modeled increasing deciles of sFlt-1, and showed a tendency towards decreasing risk of SGA as levels of sFlt-1 increased, in both unadjusted models (OR 0.92; 95%CI 0.88-0.96, P = 0.02) and models adjusted for maternal age, ethnicity, parity, BMI, height, smoking status, and hospital of delivery (OR 0.92; 95%CI 0.82-1.00, P = 0.05), suggesting the greatest risk for SGA was in the lowest decile of sFlt-1 concentration [21].

Of the other pregnancy outcomes assessed, sFlt-1 concentrations during first trimester were slightly, but not significantly higher for women who experienced a postpartum hemorrhage compared with those who did not (230 pg/mL vs. 204 pg/mL, respectively) [19]. While, compared to controls, the risk of stillbirth due to placental causes was highest in the lowest decile of sFlt-1 concentration (OR 0.71, 95% CI 0.53-0.91, p < 0.01), with the risk attenuated with increasing levels of sFlt-1 [21].

## Discussion

This systematic review provides no clear evidence for an association between sFlt-1 levels in the first trimester and adverse pregnancy outcomes. However, findings of this review were affected by methodological, biological and testing variations between studies, which highlight the need for consistent testing of new biomarkers and reporting of outcome measures.

The outcome most often investigated in these studies was preeclampsia; however, these studies do not provide a consensus as to the nature of the relationship between sFlt-1 concentrations during the first trimester of pregnancy and onset of preeclampsia. Although the majority of the studies suggest a higher level of sFlt-1 concentrations in women destined to develop preeclampsia at term, the elevated levels were not significantly different in most of the studies. Similarly, three studies that did not report the exact levels of sFlt-1 in first trimester reported no significant differences between women with and without preeclampsia [23,28-30]. This is consistent with the conclusions of Levine who found that levels didn't increase until approximately five weeks before the onset of clinical disease [5]. In contrast, preterm preeclampsia tended to be associated with lower sFlt-1 levels in first trimester. Despite these differences, one of the strengths of the preeclampsia studies was the consistency with which preeclampsia was defined and therefore differences in outcome assessment do not explain the dissimilarity of the results obtained. On the other hand, the studies were small and it is unclear whether the populations themselves are comparable. Although some studies stated that women with a history of hypertensive disorders and renal disease were excluded [16,17,20,22,24], others did not [18,23]. This may be significant as findings from the two studies that did not exclude women with previous hypertension [18,23] were in opposition to those that did [16,17,20,22,24]. The distribution of other predictors of preeclampsia such as parity, obesity, smoking and ethnicity [31], was reported in only few studies.

Similarly no conclusions could be drawn from the studies that examined the association between first trimester sFlt-1 levels and fetal growth restriction. These studies were complicated by a lack of consistency in the outcome definition and variation in testing procedures and reporting of sFlt-1 concentrations.

A number of factors, including methodological, biological and testing differences may have contributed to the variation in sFlt-1 concentrations observed in the studies included in our review. Variation in study location, characteristics of women, sample size and timing of serum screening may also impact on sFlt-1 measurements. Furthermore, the large variation in sFlt-1 levels in the control groups from the included studies also precludes comparisons. Reporting could be standardized by presenting the sFlt-1 values as multiples of the median (MoM), rather than raw concentrations, as is standard practice in Down syndrome screening [32]. This would help account for variation between maternal characteristics, gestational age, settings and testing techniques, and MoM percentile cutoffs would allow for differentiation between groups. However, only one study included in our review recognized and employed this method [21].

Given that there are no established normative levels of sFlt-1 in pregnancy, it is difficult to assess how meaningful these differences are, or to define 'abnormal" versus 'normal' concentrations. Further, there is little information about the effect of maternal factors such as smoking and body mass index on sFlt-1 levels. Small studies have reported changes in sFlt-1 concentrations between first and second trimester [18] and non-significant differences between normal pregnancies and those affected with preeclampsia [20]. However, large, prospective studies in unselected, ethnically and geographically diverse pregnant populations with serial, longitudinal measurements of sFlt-1 levels throughout pregnancy are required to assess and define the natural progression of sFlt-1 levels in pregnancy.

The variation in sFlt-1 concentrations also underscores the need to understand and ensure results are comparable between different types of biological samples. Measurement of serum and plasma may not yield similar results. With plasma, the choice of anticoagulant can influence the results; for example EDTA chelates calcium and impedes the binding of samples in testing kits which targets a calcium dependent epitope. With serum, angiogenic factors released from platelets could mask the true levels being measured. The manufacturers' package reports mean sFlt-1 concentration of 80 pg/ml (range 55-123) and 114 pg/ml (range 75-179) for EDTA-plasma and serum, respectively (n = 35 samples). If plasma samples give systematically lower concentrations than serum samples, then comparison between the two measures may not be reasonable.

Consistent testing practices across settings are also important to ensure comparability of findings. Technical differences in the storage and testing of samples may contribute to increased variability and unreliability of sFlt-1 measurements. The studies in this review varied in the length, method and temperature of sample storage, thawing of samples, duplicate testing; sensitivity of detection limits, and inter- and intra-assay coefficients of variation. Of particular importance may be temperature and length of storage, where samples stored for long periods at temperatures above -80 degrees may promote some degradation of proteins [22]. From the three studies that reported storage duration, these ranged from less than 2 years [16,22] to more than 10 years [23]. Another factor that may have contributed to the inter-study variation in the findings is the number of freeze-thaw cycles samples were exposed to. Only one study specifically stated that tested samples were only thawed once [22], and since others made no such claims, it is difficult to draw any conclusions regarding the uniformity of the measures.

The large-scale usefulness of predictive biomarkers remains unclear and such research is rarely presented in a manner that allows translation to a population level. For example, a recent study suggesting that pregnancy associated plasma protein-A (PAPP-A), an antenatal maternal serum biomarker, could be used as an early marker for pregnancies at-risk of preeclampsia reported an odds ratio of 6.6 (95% CI 2.2, 16.9), and concluded that decreased first trimester PAPP-A is a predictor of adverse pregnancy outcome [33]. However, extrapolating results from this paper suggests the marker would perform poorly at a population level, such that if 100,000 pregnancies were screened, approximately 5,000 women would be found to have abnormal PAPP-A levels, but of those, only 450 would develop preeclampsia compared to 1,425 among those with normal PAPP-A levels. Therefore, it is important to establish both the positive and negative predictive values of sFlt-1 screening before recommending its use beyond a research setting.

## Conclusions

Ideally, identification of high risk women early in pregnancy would allow for early interventions to be undertaken in hopes of reducing incidence of these outcomes. In the absence of effective interventions, a biomarker predictor of high risk patients could be used to identify women for enrolment in intervention or surveillance trials. The first trimester provides an opportunity to incorporate sFlt-1 testing into other routine testing done at the time. Screening during the 2^nd ^or 3^rd ^trimester may be too late for preventive interventions. The studies presented in this paper do not support a consensus regarding the efficacy of sFlt-1 as a predictive tool for adverse birth outcomes. However, other biomarkers used alone or in combination with sFlt-1 may be more discriminating. This review underscores the need for uniform testing and reporting to establish the utility of sFlt-1 as a first trimester screening instrument.

## Competing interests

The authors declare that they have no competing interests.

## Authors' contributions

NN, CLR, JMM, RH and AWA conceived the study, MJ and NN wrote the study proposal and MJ conducted the literature search. Data extraction was undertaken by MJ, NN, CLR and RH. MJ and NN drafted the manuscript. All authors contributed to the interpretation of findings and writing the manuscript. All authors read and approved the final draft of the manuscript.
